# 

**Published:** 1897-10

**Authors:** 


### TRI-STATE MEDICAL SOCIETY OF ALABAMA, GEORGIA AND TENNESSEE.

The Committee on Arrangements have secured the Auditorium of the "Tulane" for the Ninth Annual Meeting, to be held at Nashville, October 12th, 13th and 14th, 1897. The "Tulane" has been made the headquarters for the society during the meeting. The Address of Welcome will be delivered by Gov. Robt. L. Taylor.

This occasion promises to be the largest in the history of the society. Following is a partial list of papers promised:

President's Address—"Carcinoma of the Breast," W. F. Westmoreland, Atlanta, Ga.; "The True Physician—His Responsibilities—His Duty to his Profession, and the People," John C. LeGrand, Anniston, Ala.; "Treatment of Typhoid Fever," Jno. A. Larrabee, Louisville, Ky.; "Abortive Treatment of La Grippe," E. H. Richardson, Atlanta, Ga.; "Common Sore Throat," James H. Atlee, Chattanooga, Tenn.; "Vaccination," Seale Harris, Union Springs, Ala.; "The Pathology and Diagnosis of Early Phthisis," Llewellyn P. Barbour, Tullahoma, Tenn.; "Sero-Therapy in Tuberculosis," Paul Paquin, St. Louis, Mo.; "Importance of Early Recognition of Pleural Effusions Due to Causes Other than those

Located in the Pleuræ," Louis H. Jones, Atlanta, Ga.; "The Rational Treatment of Cancer of the Uterus," Geo. Wiley Broome, St. Louis, Mo.; "A Case of Fracture of the Skull, followed by Basilar Hemorrhage; Trephining; Recovery," Curran Pope, Louisville, Ky.; "The Relation of the Cause to the Immediate and Remote Results of Fracture," R. M. Cunningham, Birmingham, Ala.; "Is Cancer Contagious?" E. Mather, Patterson, N. J.; "Stimulants and Narcotics in Obstetrics and Gynecology," R. R. Kime, Atlanta, Ga.; "Surgical Shock," Gilbert I. Cullen, Cincinnati, O.; "Cystitis; Its Course and Treatment," W. L. Nolen, Chattanooga, Tenn.; "Stricture of the Urethra and its Treatment," Wm. R. Blue, Louisville, Ky.; "Operative Treatment in Enlarged Prostate," H. H. Grant, Louisville, Ky.; "Ablation of the Scrotum for Conditions other than Varicocele," W. S. Goldsmith, Atlanta, Ga.; "The Treatment of Cancer of the Rectum," J. M. Mathews, Louisville, Ky.; "Burns and Scalds," G. A. Baxter, Chattanooga, Tenn.; "Metatarsalgia, or Morton's Painful Toe," Geo. S. Brown, Birmingham, Ala.; "The Application of Plaster Jackets and Dressings," F. B. Sloan, Cowan, Tenn.; "Surgery of the Stomach," H. Berlin, Chattanooga, Tenn.; "Report of Operative Cases (Brain)," S. G. Courtney Pinckney, Atlanta, Ga.; "Fracture of the Elbow," B. G. Copeland, Birmingham, Ala.; "Epiplocele, Report of a Case," J. W. Griggs, West Point, Ga.; "Appendicitis," G. Manning Ellis, Chattanooga, Tenn.; "The Evolution of the Treatment of the Stump, in Operations for Appendicitis," W. D. Haggard, Jr., Nashville, Tenn.; "Some of the Mammoth Ovarian Tumors of Surgical History," A. M. Cartledge, Louisville, Ky.; "Cases of Ectopic Gestation, Operated on by the Vaginal Route," W. E. B. Davis, Birmingham, Ala.; "The Technique in Hysterectomy for Uterine Myomata," W. H. Wathen, Louisville, Ky.; "Hysterectomy," Louis Frank, M.D., Louisville, Ky.; "Hysterectomy in the

Treatment of Pelvic Diseases," Geo. R. West, Chattanooga, Tenn.; "Flap Operation for Atresia Vaginæ," Geo. H. Noble, Atlanta, Ga.; "Meningitis in Children," J. W. Russey, Chattanooga, Tenn.; "Mouth Breathing in Children and its Dangers," Wm. Cheatham, Louisville, Ky.; "Injurious Results of Incompetent Refraction Work," Frank Sims, Atlanta, Ga.; "The Causes, Diagnosis, and Prognosis, of Valvular Diseases," Hazle Padgett, Columbia, Tenn.; "Rheumatism as an Etiological Factor in Cardiac Diseases," S. W. Fain, Chattanooga, Tenn.; Subject to be announced, A. W. Calhoun, Atlanta, Ga.; "Various Treatments of Epithelioma," M. B. Hutchins, Atlanta; "Leukæmia and Report of a Case of Hodgkin's Disease," A. W. Mardis, Lebanon, Ohio; "Report of Some Cases of Abdominal Surgery," James M. Black, Knoxville; "Posterior Urethritis," R. H. Tatum, Chattanooga; "Chloroform," Cooper Holtzclaw, Chattanooga; "Stricture of the Nasal Duct," B. F. Travis, Chattanooga; "Cataract Operations," Jas. Moores Ball, St. Louis.

---

### NOTICE TO SUBSCRIBERS.

To meet the convenience of the country practitioners it has long been our custom to leave their subscription accounts open until October 1st of each year. Many then pay their bills. We thank them again for being willing to pay for what they get. Those who do not pay promptly are either negligent, careless or unable to pay. The negligent and careless should pay up. Those who are unable to pay are welcome to *The Journal*. Those who prefer manufacturers' literature, sample copies, or an eclectic journal, if it is cheaper, ought to stop a journal which they do not pay for.

We send you the regular October statement. Any errors will be gladly corrected. We give you a good journal, and it is worth to you the subscription price—or not worth your receiving.

